# A 1-aminocyclopropane-1-carboxylic-acid (ACC) dipeptide elicits ethylene responses through ACC-oxidase mediated substrate promiscuity

**DOI:** 10.3389/fpls.2022.995073

**Published:** 2022-09-12

**Authors:** John Vaughan-Hirsch, Dongdong Li, Albert Roig Martinez, Stijn Roden, Jolien Pattyn, Shu Taira, Hitomi Shikano, Yoko Miyama, Yukari Okano, Arnout Voet, Bram Van de Poel

**Affiliations:** ^1^Division of Crop Biotechnics, Department of Biosystems, University of Leuven, Leuven, Belgium; ^2^College of Agriculture and Biotechnology, Zhejiang University, Hangzhou, China; ^3^Division of Biochemistry, Molecular and Structural Biology, Department of Chemistry, University of Leuven, Leuven, Belgium; ^4^Department of Agriculture, Fukushima University, Fukushima, Japan; ^5^KU Leuven Plant Institute, University of Leuven, Leuven, Belgium

**Keywords:** ethylene, 1-aminocyclopropane-1-carboxylic acid (ACC), di-ACC, ACC oxidase (ACO), non-proteinogenic amino acid, dipeptide, enzyme promiscuity

## Abstract

Plants produce the volatile hormone ethylene to regulate many developmental processes and to deal with (a)biotic stressors. In seed plants, ethylene is synthesized from 1-aminocyclopropane-1-carboxylic acid (ACC) by the dedicated enzyme ACC oxidase (ACO). Ethylene biosynthesis is tightly regulated at the level of ACC through ACC synthesis, conjugation and transport. ACC is a non-proteinogenic amino acid, which also has signaling roles independent from ethylene. In this work, we investigated the biological function of an uncharacterized ACC dipeptide. The custom-synthesized di-ACC molecule can be taken up by Arabidopsis in a similar way as ACC, in part *via* Lysine Histidine Transporters (e.g., LHT1). Using Nano-Particle Assisted Laser Desoprtion/Ionization (Nano-PALDI) mass-spectrometry imaging, we revealed that externally fed di-ACC predominantly localizes to the vasculature tissue, despite it not being detectable in control hypocotyl segments. Once taken up, the ACC dimer can evoke a triple response phenotype in dark-grown seedlings, reminiscent of ethylene responses induced by ACC itself, albeit less efficiently compared to ACC. Di-ACC does not act *via* ACC-signaling, but operates *via* the known ethylene signaling pathway. *In vitro* ACO activity and molecular docking showed that di-ACC can be used as an alternative substrate by ACO to form ethylene. The promiscuous nature of ACO for the ACC dimer also explains the higher ethylene production rates observed *in planta*, although this reaction occurred less efficiently compared to ACC. Overall, the ACC dipeptide seems to be transported and converted into ethylene in a similar way as ACC, and is able to augment ethylene production levels and induce subsequent ethylene responses in Arabidopsis.

## Introduction

The gaseous plant hormone ethylene plays an important role in plant developmental processes such as germination, vegetative growth, climacteric fruit ripening, senescence and abscission (Abeles et al., 1992). Ethylene is also involved in responses toward biotic and abiotic stressors (Chen et al., 2022). Since ethylene gas is inert and rapidly diffuses from its site of synthesis, regulation of ethylene production must take place at the level of its biosynthesis pathway (Pattyn et al., 2021). In embryophytes, ethylene is synthesized in two dedicated steps, from the methionine derivative S-adenosyl-L-methionine (SAM). First, SAM is converted to 1-aminocyclopropane-1-carboxylic acid (ACC) catalyzed by ACC synthase (ACS) (Adams and Yang, 1977, 1979; Boller et al., 1979). ACC is then oxidized to form ethylene *via* the action of ACC oxidase (ACO) (Hamilton et al., 1991; Ververidis and John, 1991; Houben and Van de Poel, 2019). Several ACC conjugates have been described, which may limit the pool of available ACC for ethylene production (reviewed by Pattyn et al., 2021). These include ACC conjugated with a malonyl group (MACC), a jasmonyl group (JA-ACC) and a γ-glutamyl group (GACC) (Van de Poel and van der Straeten, 2014). It is likely there are other ACC conjugates which are only present in certain species, certain tissues or under certain conditions. More recently, ACC has also been shown to function as a signaling molecule independent of its role in ethylene production (Polko and Kieber, 2019; Li et al., 2022). Understanding the role of ACC and its derivatives is therefore of interest, as they may converge on ethylene-dependent and ethylene-independent signaling pathways.

ACC is one of several non-proteinogenic amino acids (NPAAs) that are synthesized in plants (Fowden, 1981). Other examples of NPAAs are ornithine, citrulline, arginosuccinate, homoserine, homocysteine, and cystathionine (Jander et al., 2020). Many NPAAs function as metabolic intermediates, whereas others function as signaling molecules. ACC belongs to both groups, since it is a direct precursor to ethylene but also has independent signaling functions. In this respect, ACC shows similarity to γ-aminobutyric acid (GABA), another NPAA. GABA is found in bacteria, fungi, plants and animals, and in plants functions in both metabolism and signaling (Bouché and Fromm, 2004; Ramesh et al., 2015, 2016). In animals GABA functions as a major inhibitory neurotransmitter, and is also used as a substrate in the formation of homocarnosine, a dipeptide formed from GABA and L-histidine (Crush, 1970). Homocarnosine has distinct roles from GABA and is absent in plants (Bauer, 2005). Similarly, ACC has also been shown to act as a partial agonist to the mammalian neuron NMDA receptors (Inanobe et al., 2005). Whether ACC also occurs as a dipeptide remains uninvestigated. Several dipeptide molecules have been discovered in plants, but these are in large part synthesized *via* protein catabolism (Calderan-Rodrigues et al., 2021; Thirumalaikumar et al., 2021). Formation of NPAA dipeptides however, must occur *via* dedicated enzymes, as they cannot be incorporated into peptides *via* mRNA translation.

Similarly to dipeptides, several small signaling peptides composed of proteinogenic amino acids have been discovered in plants (Irving and Gehring, 2012). These belong to two major groups: small post-translationally modified peptides (which are derived from processing of longer precursors to peptides under 20 amino acids in length), and Cys-rich peptides (which are not post-translationally processed, and are characterized by a conserved N-terminal signal peptide and a Cys-rich C-terminal, with a length of under 160 amino acids) (Murphy et al., 2012). These peptides have many crucial roles in plant development and stress responses, including plant defense (Murphy et al., 2012; Takahashi et al., 2019). Probably the best studied of these small signaling peptides include the CLE (CLAVATA3/Embryo Surrounding Region-Related) family, which are involved in a huge range of developmental processes (Fletcher, 1999; Yamaguchi et al., 2016). These small signaling peptides differ from the NPAA peptides in that they are direct products of mRNA translation or protein catabolism.

In this work, we investigate the putative role of an ACC dipeptide (di-ACC) in Arabidopsis and *Marchantia polymorpha*. We revealed that a dimer containing two ACC molecules fused *via* a peptide bond can exert ethylene responses in Arabidopsis, albeit less efficiently compared to the ACC monomer. The ACC dipeptide was taken up by roots and transported in a similar way to ACC, despite di-ACC not occurring naturally in Arabidopsis. Fed di-ACC is catabolized *via* ACO substrate promiscuity which converts di-ACC into ethylene.

## Materials and methods

### Di-ACC synthesis and purification

Di-ACC was synthesized by GenScript Biotech and confirmed by mass spectrometry and HPLC to have a purity of 95.6%. Because the synthesis used Fmoc-ACC (Fluorenylmethoxycarbonyl-ACC) as a building block, which may be converted to ACC *in planta*, we further purified the di-ACC by HPLC to eliminate any residual ACC contamination. Di-ACC fractions in 0.1% TFA were collected after passing over a Hypercarb^TM^ column (Thermo Scientific) using acetonitrile as solvent (Supplementary Figure S1A), and purified samples run again on the same column to confirm elimination of contaminant peaks (Supplementary Figure S1B). ACC was run independently to enable calculation of the retention time.

### Plant material, growth conditions and phenotyping

*Arabidopsis thaliana* ecotype Col-0 was used as wild-type control, the *etr1-1* (AT1G66340; N237) EMS mutant and *lht1-5* (AT5G40780; SALK_115555C) mutant were obtained from NASC. Seeds were surface sterilized by incubation with 70% ethanol (v/v) for 2 mins, followed incubation in 5% bleach (NaOCl) (v/v) for 5 mins. Seeds were then washed in sterile H_2_O and plated in Petri dishes containing 0.5 × Murashige and Skoog (MS) basal salt medium with 1% plant agar (pH 5.7). Media was supplemented with ACC or di-ACC at concentrations indicated. Seeds were imbibed at 4°C for 3 days before transfer to the growth room. For triple response phenotyping and ethylene measurements, seedlings were grown for 4 days in the dark at 21°C before measurements. Root and shoot length was quantified using ImageJ (http://rsbweb.nih.gov/ij/) from photographs. For Nano-PALDI MSI, seedlings were grown for 10 days in a growth chamber (16 h light/8 h dark; 21°C) before treatment with ACC or di-ACC. For triple response assays, plants were treated with 0.5, 1 or 20 μM ACC and increasing concentrations of di-ACC of 0.5, 1, 2, 5, 10, 20, 50 μM. For ethylene measurements, plants were treated with 0.1, 0.5 and 1 μM ACC, and 2 and 10 μM di-ACC.

*Marchantia polymorpha* Australian ecotype (MEL accession) wild type (“WT1” female) and the Mp*ein3* mutant used in this study were previously described in Li et al. (2020). Single gemma were plated on solid half-strength Gamborg's B5 salts medium (pH 5.5) supplemented with 0.2% sucrose and 1% plant agar. ACC (100 μM) or di-ACC (10 and 100 μM) was added to the media prior to plating. Plants were grown at 21°C under sunlight-mimicking LED lights at 200 μmol.m^−2^.s^−1^ for a 16/8 h day/night regime. Plants were imaged after 15 days under a stereoscopic microscope (Olympus SZX9) equipped with a camera (Toupcam, Touptek Photonics) or photographed directly with a digital camera (Nikon D3400). The two-dimensional plant area (thallus size) was quantified using ImageJ.

### Ethylene measurements

For *in planta* ethylene measurements, 1 ml of headspace gas from seedlings grown in 4 ml airtight GC vials was injected into a gas chromatograph (Shimadzu GC2014) as described in Houben et al. (2022).

### Nano-PALDI MS imaging

Nano-PALDI MSI was carried out as previously described (Shiono et al., 2017; Ortigosa et al., 2022; Taira and Shiono, 2022), but with a few modifications for small tissues. Briefly, 10 day-old seedlings of Arabidopsis ecotype Col-0 were incubated in 2 μM ACC or di-ACC overnight. The seedlings were then embedded in Super Cryoembedding Medium (SCEM, Leica) and frozen in liquid nitrogen. The specimen block was cut into 10 μm sections using a cryostat (NX-70, Thermo Fisher Scientific, USA) set at −23°C in the chamber and at −25°C on the object holder. The sections were mounted on slides coated with indium tin oxide (ITO) (Bruker Daltonik GmbH). Optical images of sections were obtained by a virtual slide scanner (NanoZoomer-SQ, Hamamatsu Photonics) before analysis by Nano-PALDI-MSI.

For nano-PALDI-MSI, iron oxide-based nanoparticles (Fe-NPs) were prepared by stirring aqueous solutions of FeCl_2_·4H_2_O (5 ml, 100 mM), and 3-aminopropyltriethoxysilane (5 ml; γ-APTES) at room temperature for 1 h. The resulting precipitate was washed several times with ultrapure water and then suspended in methanol. One mg Fe-NPs was suspended in 1 ml methanol and sprayed on tissue sections on ITO-coated glass slides with an airbrush (nozzle caliber, 0.2 mm). To obtain MS images, each data point on the section were irradiated with 200 laser shots in the positive ion detection mode of the mass spectrometer. Only signals between 80 and 500 *m/z* were analyzed to detect the correlated ACC (*m/z* 102.1) and ACC dipeptide (*m/z* 185.0) as protonated ions, respectively. For each section, ~100,000 data points were obtained, 5 μm apart. The MS image was reconstructed from the obtained MS spectra with a mass bin width of *m/z* ± 0.05 from the exact mass using flexImaging 5.0 (Bruker Daltonik GmbH). The accurate mass of the ions was used for image generation, and mass accuracy and root-mean-square error (RMSE) were automatically calculated by the imaging software to avoid false-positive signals.

### ACO2 expression and purification

The coding sequence of AtACO2 (At1g62380) was amplified and cloned into the pET28a bacterial expression vector, and transformed into E. coli strain BL21 (DE3). Starter cultures were grown in 5 ml LB media with 50 μg/ml kanamycin and chloramphenicol overnight at 37°C shaking at 200 rpm. Starter cultures were used to inoculate 2 L cultures, which were grown at 37°C shaking at 100 rpm until an OD600 of 0.8 was reached, measured using an Ultrospec 10 Cell Density meter (Amersham Biosciences). Cultures were then cooled on ice for 30 mins, protein expression induced by adding IPTG to a final concentration of 0.5 mM, and then grown overnight at 18°C shaking at 100 rpm. Cells were pelleted by centrifugation at 5,500 rpm for 30 mins at 4°C, resuspended in lysis buffer, rotated at room temperature for 30 mins, and sonicated with a Branson 450 sonifier (VWR) with a power of 3 and duty cycle of 80, for six cycles of 1 min sonication and 2 mins rest on ice. Cell debris was removed by centrifugation for 40 min at 13,000 rpm at 4°C, and filtration of the supernatant through a 0.45 μm filter. AtACO2 was first purified by IMAC chromatography (Qiagen Ni-NTA, 5–250 mM Imidiazole, 200 mM NaCl, 50 mM NaH_2_PO_4_, 1 mM DTT, pH 8) followed by anion exchange chromatography on an Äkta^*TM*^ Prime Plus chromatography system (GE Healthcare) fitted with a 5 ml HiTrap^*TM*^ Q HP column (Cytiva) (25–1,000 mM NaCl, 50 mM NaH_2_PO_4_, 1 mM DTT, pH 8). SDS PAGE confirmed protein purity at every step. Protein concentration was calculated using a NanoDrop^*TM*^ 2000 spectrophotometer (Thermo Scientific).

### *In vitro* ACO activity assay

For *in vitro* ACO activity measurements (Van de Poel et al., 2014), recombinant purified AtACO2 (5 μg) was incubated in freshly prepared activity buffer containing 50 mM MOPS, 5 mM ascorbic acid, 20 mM sodium bicarbonate, 10% glycerol, 0.1 mM DTT and the mentioned ACC or di-ACC concentration. The reaction was incubated in 4 ml airtight GC vials for 60 min at 30°C while shaking. One ml of headspace was sampled and analyzed for ethylene content using gas chromatography as described above.

### Modeling di-ACC-ACO interaction

The di-ACC-ACO2 complex was modeled in Molecular Operating Environment Software (MOE) (Chemical Computing Group ULC), using crystal structures of Arabidopsis ACO2 (PDB 5GJ9) and the ACC-bound ACO of *Petunia hybrida* (PDB 5TCV) (Sun et al., 2017). Amber10 was used for complex simulation and calculation of partial charges (Case et al., 2008).

### Statistical analyses

Data was analyzed in Graphpad Prism version 8.0.2. The Shapiro-Wilk normality test was performed, and normally distributed data tested by ordinary one-way ANOVA and a Tukey *post-hoc* test. When the assumption of normality was not met, a non-parametric Kruskal–Wallis one-way ANOVA test was used. For ethylene production, biological variation between data sets was high, so a Welsh ANOVA with Holm-Sidak's multiple comparisons test was used.

## Results

### ACC dimer induces triple response phenotype reminiscent of ACC treatment

**Figure 1:**
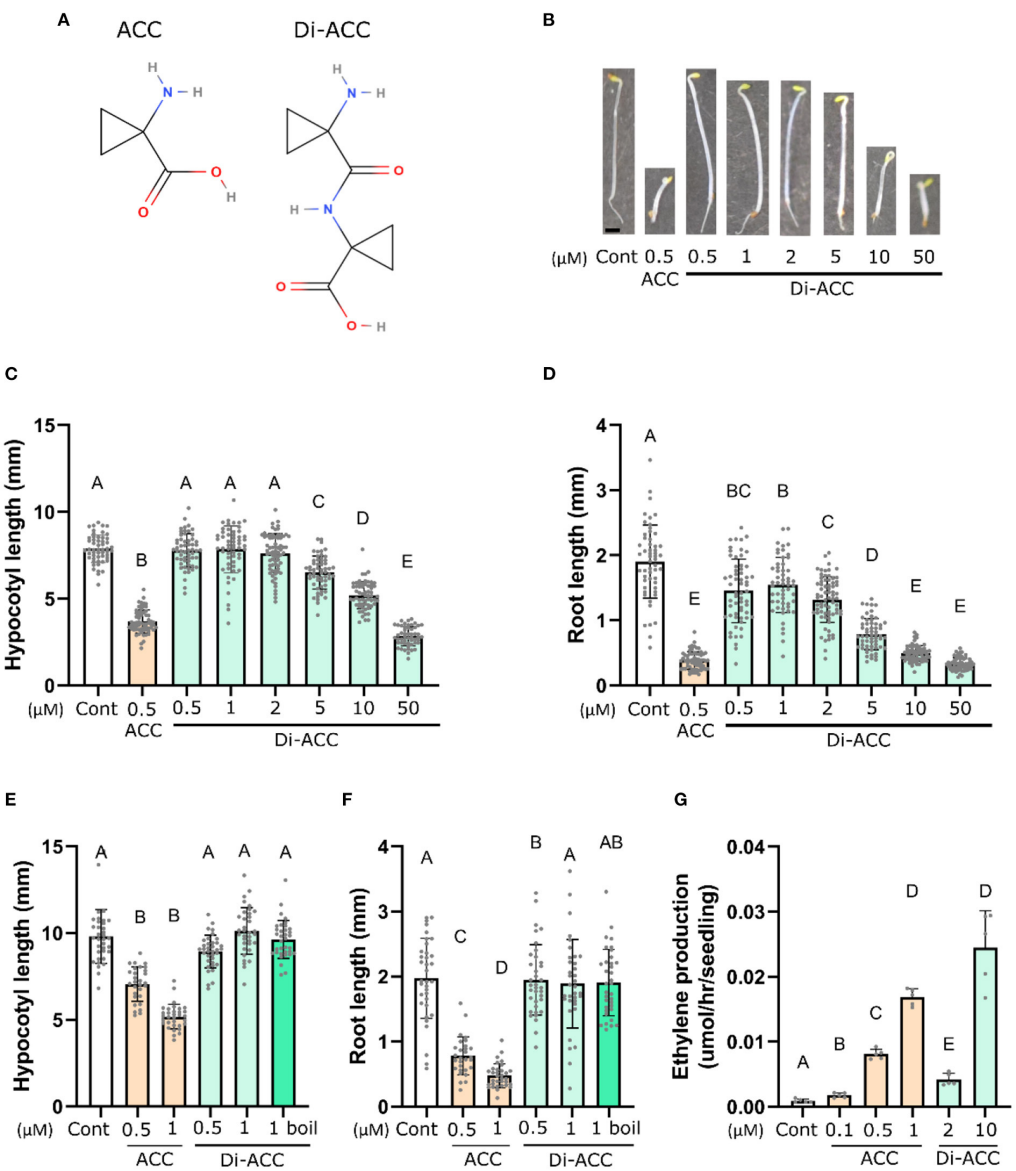
ACC dimer induces the triple response phenotype in Arabidopsis. **(A)** The molecular structures of ACC and its dipeptide di-ACC. **(B)** Representative images and **(C,D)** quantification of hypocotyl **(C)** and root length **(D)** of 4-day-old dark-grown seedlings grown on medium containing ACC or di-ACC (*n* ≥ 54). **(E,F)** Quantification of hypocotyl **(E)** and root length **(F)** in a triple response assays after boiling di-ACC for 1 h prior to addition to the culture media (*n* ≥ 29). **(G)** *In planta* ethylene production of 4-day-old dark-grown seedlings treated with ACC or di-ACC (*n* = 5). Scale bar in **(B)** is 1 mm.

In analogy to the existence of other non-proteinogenic dipeptides in plants, we hypothesized that ACC may also exist as a dipeptide (Figure 1A), and as such may represent a novel ACC derivative which could influence ethylene biosynthesis or ACC signaling. To investigate this possibility, we had di-ACC custom synthesized, followed by HPLC purification to remove residual trace amounts of ACC from the synthesis process (Supplementary Figure S1). First, we examined whether di-ACC is able to evoke an ethylene response in plants, deploying the characteristic triple response assays of 4-day-old dark-grown Arabidopsis seedlings. We observed that di-ACC induces shorter roots and hypocotyls, and an exaggerated apical hook, reminiscent of ethylene responses (Figures 1B–D). However, these effects were only observable at higher di-ACC concentrations as compared with ACC treatment, with hypocotyl shortening only seen with di-ACC treatment of 5 μM or higher.

Since the di-ACC is a newly synthesized molecule, we have no data on its stability. It is therefore possible that the ACC dipeptide was broken down during the course of experiments (for example by thermal instability) and release ACC, leading to the observed triple response phenotype (Figures 1B–D). To rule out di-ACC instability, we performed a triple response assay including di-ACC which had been boiled for 1 h prior to making media plates (Figures 1E,F). Hypocotyl and root length of seedlings treated with boiled di-ACC were not significantly different from those treated with the same concentration of fresh di-ACC, indicating that phenotypes seen with higher di-ACC concentrations are not due to release of free ACC from degradation of di-ACC. We also included boiled ACC as a control, which also shows similar activity as fresh ACC (Supplementary Figure S2), indicating that both ACC and di-ACC are thermostable molecules.

To investigate the observation that di-ACC evokes an ethylene response through the synthesis of ethylene (similar as ACC), we measured ethylene production of 4-day-old dark-grown seedlings treated with ACC and di-ACC (Figure 1G). This revealed that a di-ACC treatment is able to elevate ethylene production levels, albeit a ten-fold higher concentrations of di-ACC is needed to get similar levels of ethylene compared to ACC. This is similar to the requirement for higher concentrations of di-ACC to generate triple response phenotypes (Figures 1B–D), as compared to ACC. Overall, this proves that di-ACC is converted into ethylene *in planta*.

### ACC dimer is taken up in part by LHT1 and triggers ethylene responses *via* the ethylene signaling pathway

**Figure 2:**
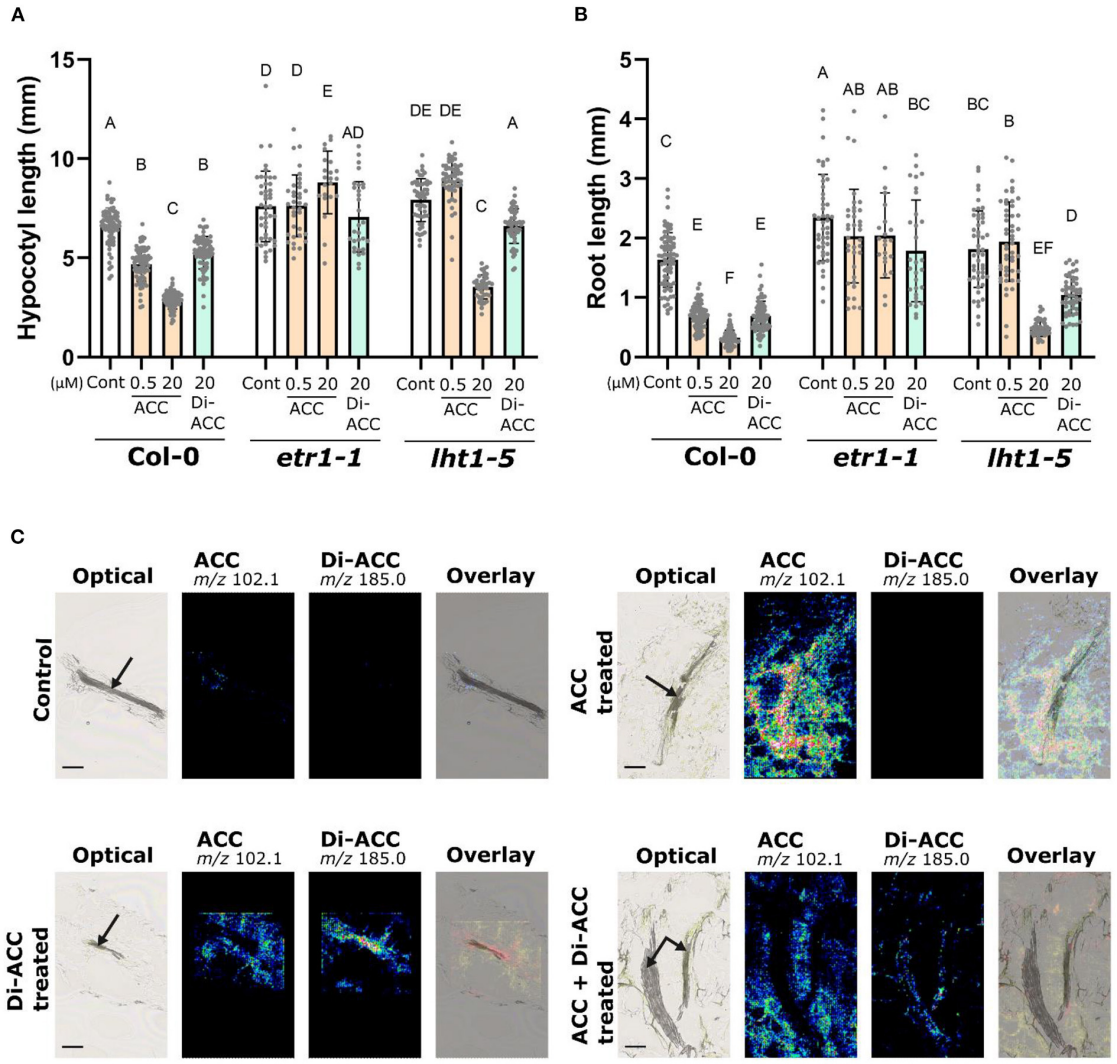
ACC dimer induces triple response phenotype via ethylene signaling and shows an LHT1-dependend mobility. **(A)** Hypocotyl and **(B)** root length of 4-day-old dark-grown Col-0 wild-type, *etr1-1* and *lht1-5* seedlings after growth with ACC or di-ACC (*n* ≥ 22). **(C)** Nano-PALDI MS images of hypocotyl sections of plants treated overnight with 2 μM ACC, di-ACC, or both. Panels show optical images, ACC signal (*m/z* 102.1), di-ACC signal (*m/z* 185.0) and an overlay. The overlay for control and ACC treated plants includes the optical image and the ACC signal, and for di-ACC and ACC + di-ACC treated plants includes the optical image, ACC and di-ACC signals (yellow for ACC and red for di-ACC). The vascular tissue is indicated with arrows.

Figure 1 showed that di-ACC is able to augment ethylene production and evoke ethylene responses in Arabidopsis seedlings (reviewed by Li et al., 2022). To validate if the triple response phenotype of di-ACC treated plants (Figures 1C,D) is ethylene-dependent, we phenotyped the ethylene insensitive mutants *etr1-1*. The *etr1-1* mutant shows complete insensitivity toward both ACC and di-ACC for both the root and the hypocotyl (Figures 2A,B), suggesting that the triple response evoked by di-ACC occurs *via* ethylene signaling by the ethylene receptor. In order to determine if the ACC dipeptide is taken up and transported in a similar way as ACC, we evaluated the triple response phenotype of the *lht1-5* mutant, harboring a reduced ACC uptake due to a dysfunctional Lysine Histidine Transporter1 (LHT1) (Shin et al., 2015). In the *lht1-5* mutant, the di-ACC treatment causes a partial triple response phenotype, with a reduced root and hypocotyl length, but not as severe as compared to the Col-0 background (Figures 2B,C). This indicates that di-ACC, similar as ACC, is taken up in part *via* LHT1, but also that di-ACC can still be taken up by other transporters.

To visualize ACC and di-ACC *in planta* we made use of Nano Particle-Assisted Laser Desorption/Ionization mass spectrometry imaging (Nano-PALDI MSI), which has recently been used to identify the spatial distribution of hormones and metabolites in plant tissue sections (Shiono et al., 2017; Ortigosa et al., 2022; Taira and Shiono, 2022). Ten day-old Arabidopsis seedlings were treated with 2 μM ACC, di-ACC or both overnight, before hypocotyl sections were analyzed using Nano-PALDI MS imaging (Figure 2C). Untreated control samples show a weak signal for ACC in tissue mainly surrounding of the vasculature, whereas there is no detection of the di-ACC, suggesting di-ACC is naturally not present in Arabidopsis hypocotyls (or not detectable). When ACC was fed, a strong ACC mass signal (*m/z* 102.1) was seen in all tissues, while no di-ACC mass signal (*m/z* 185.0) was observed in the sample. A di-ACC treatment showed that di-ACC was taken up by the sample, and largely confined to the vascular tissue. When both ACC and di-ACC were fed, ACC was observed much more broadly in the surrounding tissues, while di-ACC seemed more restricted to the vasculature. This tissue-specific accumulation suggests that di-ACC is less efficiently transported from the vasculature to the surrounding tissues compared to ACC. Furthermore, we observed a relatively lower signal intensity for di-ACC compared to ACC, suggesting that di-ACC has a lower affinity for ACC transporters, and consequently ACC can outcompete di-ACC for uptake.

In di-ACC treated plants, the ACC signal may look more intense compared to control plants, suggesting di-ACC may be catabolized to ACC. However, it should be noted that comparisons of Nano-PALDI MS images are semi-quantitative, because each signal value was derived from the relative intensity normalized by the highest intensity spot per slide. Therefore we cannot be certain that catabolism of di-ACC to ACC takes place *in planta*. Nonetheless, the restricted spatial distribution of the di-ACC is a clear indicator of its reduced mobility *in planta* compared to ACC. Additionally, the requirement for higher concentrations of di-ACC to evoke the triple response (see Figures 1B–D), could partially be explained by a reduced uptake and a reduced mobility of the ACC dimer *in planta*.

### ACC dimer does not activate ethylene-independent ACC signaling in *Marchantia*

**Figure 3:**
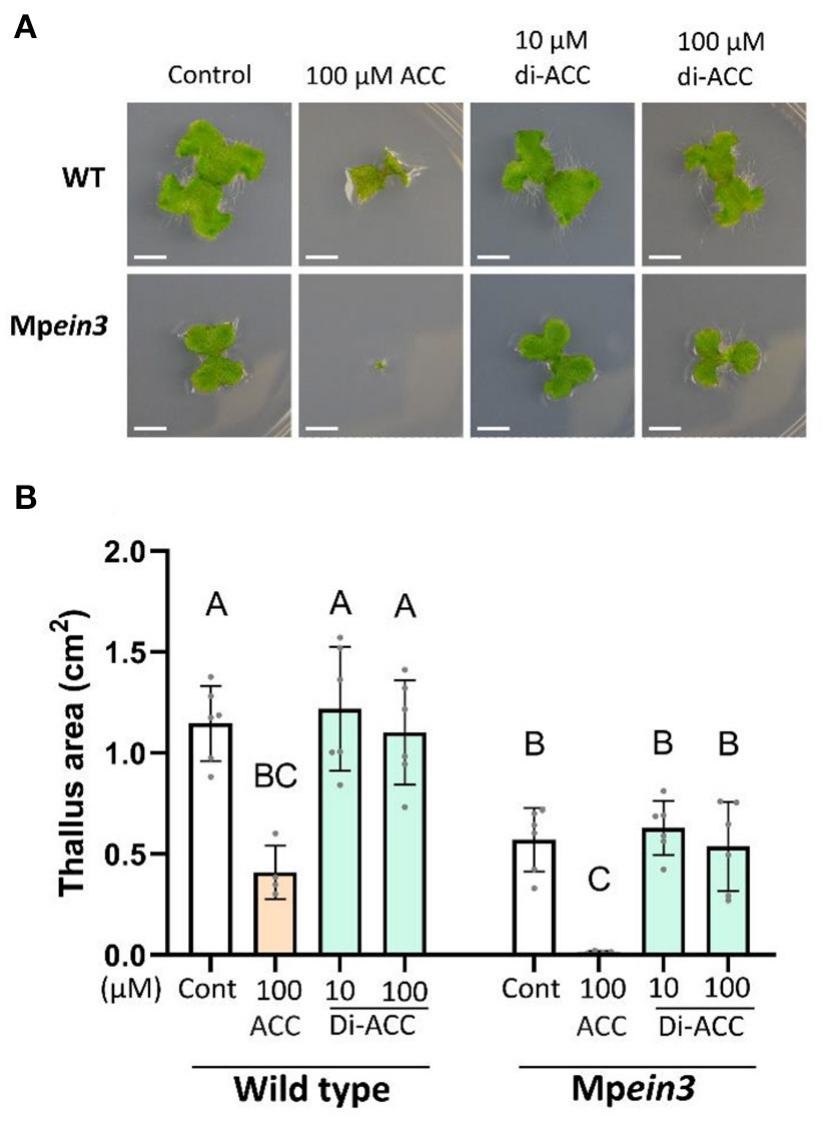
The ACC dimer does not induce ethylene-independent ACC signaling in *Marchantia*. **(A)** Representative images and **(B)** quantification of *Marchantia polymorpha* ground cover in WT and Mp*ein3* plants, after ACC or di-ACC treatment (*n* ≥ 4). Scale bars are 5 mm.

To rule out that di-ACC is also able to function *via* an ethylene-independent ACC signaling pathway, we tested the response of *Marchantia polymorpha* to the ACC dimer (Figure 3). The *Marchantia* genome does not encode any ACOs, and therefore ACC is not used as an efficient precursor for ethylene production. However, an ACC treatment results in an ethylene-independent reduction of the thallus area (Li et al., 2020), a phenotype which is amplified in the Mp*ein3* mutant. Treatment of *Marchantia polymorpha* with di-ACC did not reduce thallus area in either background, while an ACC treatment resulted in a much smaller thallus area (Figure 3). This result indicates that di-ACC is not able to function in the ethylene-independent ACC signaling pathway in *Marchantia*.

### ACC dimer increases ethylene biosynthesis directly *via* ACO activity

**Figure 4:**
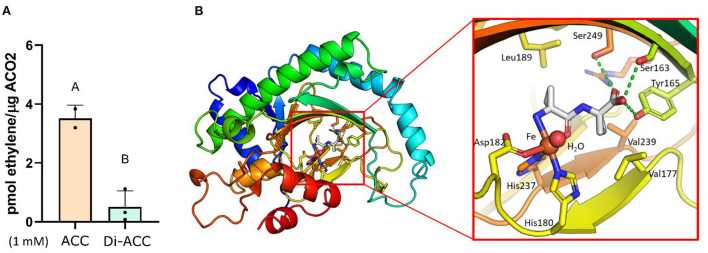
Di-ACC acts as a substrate for ACO to form ethylene, with lower efficiency than ACC. **(A)** *In-vitro* ethylene production using recombinant purified AtACO2 with ACC or di-ACC as substrate (*n* = 3). **(B)** Simulated binding of di-ACC in the catalytic core of AtACO2. The right panel shows a close-up of the catalytic site in which the ACC dimer is superimposed with free ACC (shown in gray with blue amino groups and red carboxyl groups) and the Fe-metal co-factor in red. Important residues that facilitate substrate docking are indicated with amino acid numbers. Hydrogen bonds are shown as green dotted lines.

Having ruled out the possibility that di-ACC associated phenotypes are caused by di-ACC breakdown into ACC during treatment (Figures 1E,F), and ethylene independent ACC signaling (Figure 3), it remains unknown how di-ACC is able to elevate ethylene biosynthesis (Figure 1G) and subsequently activate the ethylene signaling pathway (Figure 2) leading to ethylene responses in Arabidopsis (Figures 1A–C). It is possible that di-ACC is catabolized to ACC *in planta* (by an unknown peptidase) which can subsequently be processed to form ethylene. Alternatively, it is possible that di-ACC is used directly by ACO as a substrate for ethylene synthesis. To address this question, we measured *in vitro* ethylene production, using recombinant purified AtACO2. In this system, we were able to measure low levels of ethylene production when di-ACC was fed (Figure 4A), but could not detect any ethylene in control samples without any substrate (not shown). Interestingly, *in vitro* ethylene production levels for di-ACC were around 4 times lower compared to feeding the same concentration of ACC. This result indicates that ACO2 is able to use di-ACC directly as a substrate for ethylene synthesis, albeit with a lower efficiency compared to ACC.

To explore how di-ACC can act as a substrate for ACO, we examined the molecular docking prediction between di-ACC and ACO. The complex was modeled using the Arabidopsis ACO2 structure, and the ACC-bound complex of an ACO from *Petunia hybrida* (Sun et al., 2017). Superimposition of the di-ACC on the mono-ACC followed by a short local energy optimization revealed no steric hindrance, while the free carboxyl end of di-ACC is compensated by the hydrogen bonds formed by Ser163, Tyr165 and Ser249. The second cyclopropyl group sits sandwiched between Val117 and Val239. The Fe-bound water molecule required for catalytic activity is also not influenced by the di-ACC placement. The lower catalytic efficiency of di-ACC vs. ACC in our *in vitro* assay, could be explained by the lower affinity of the metal ion for the coordination oxygen, which has a calculated partial charge of −0.9 in the carboxyl free (ACC) compared to −0.57 in case of the amide-form (di-ACC), as calculated by the Amber10-EHT forcefield (Case et al., 2008).

This suggests that di-ACC is able to bind to the catalytic site of AtACO2, albeit with a lower efficiency than ACC. The higher concentrations of di-ACC needed to evoke a similar triple response phenotype compared to ACC, is likely due to reduced affinity of di-ACC for ACO, in combination with a reduced uptake and *in planta* mobility of the ACC dimer.

## Discussion

As major regulators of plant development and stress response, the homeostasis of plant hormones must be carefully controlled. This can occur at the level of biosynthesis, downstream signaling, or by direct modification of the plant hormone. One common modification is the conjugation of hormones with amino acids, which can be used to inactivate or target a hormone for degradation or storage (Jez, 2022). Sometimes amino acid conjugated hormones retain biological activity. For example, conjugation of isoleucine (Ile) to jasmonic acid (JA) yields JA-Ile, one of the most bioactive jasmonates (Staswick et al., 2002; Fonseca et al., 2009). Conjugation with different amino acids can also lead to different responses. Conjugation of auxins (IAA) with aspartate or glutamate inactivates them, whereas conjugation with alanine or leucine is used for storage (Ludwig-Müller, 2011). Often, JA or IAA amino acid conjugation is conducted by a family of GRETCHEN HAGEN 3 (GH3) acyl acid amido synthetases (Jez, 2022). Salicylic acid and cytokinins have also been identified in the form of amino acid conjugates (Westfall et al., 2016; Rekhter et al., 2019; Torrens-Spence et al., 2019). Although amino acid conjugation of abscisic acid (ABA) has not yet been identified, a study of synthetic ABA-amino acid conjugates revealed some conjugates are hydrolyzed to release free ABA, yet being species dependent (Todoroki et al., 2011).

As a volatile gas, ethylene itself is not known to be modified. However, conjugation of its precursor ACC can restrict ethylene production. Currently three conjugates of ACC have been described: MACC, JA-ACC and GACC, all of which are unable to be converted to ethylene (Van de Poel and van der Straeten, 2014). Although currently unknown, it is possible that conjugation of ACC with other amino acids exist, to form dipeptides. Several dipeptides have been characterized in plants, many of which have signaling or metabolic roles (Calderan-Rodrigues et al., 2021; Moreno et al., 2021). Here, we found that the synthetic ACC dipeptide is able to evoke an ethylene response in Arabidopsis (Figure 1), independent of ACC signaling (Figure 3).

## Transport of ACC dimer is reminiscent of ACC and partially mediated by LHT1

In Arabidopsis, ACC is taken up by the broad-specificity amino acid transporter LHT1 (Shin et al., 2015). Interestingly, LHT1 is also able to transport the dipeptide GABA (Hirner et al., 2006), and as such is a good candidate for a transporter of the ACC dipeptide (Hirner et al., 2006). Feeding di-ACC to the *lht1-5* mutant resulted in a less severe triple response phenotype compared to Col-0 (Figures 2A,B), suggesting that while LHT1 is able to transport the ACC dimer, there may also be some redundancy with other transporters. Di-ACC may also be transported by LHT2, which was recently shown to function in ACC uptake (Choi et al., 2019), and it is possible there are other unknown amino acid transporters also able to transport di-ACC.

LHT1 and similar amino acid transporters may be responsible for the competitive inhibition of di-ACC uptake by ACC, as observed by our nano-PALDI MS imaging in plants treated with a combination of ACC and di-ACC (Figure 2C). While LHT1 and LHT2 are able to transport a broad range of amino acids, they do so with different affinities dependent on the amino acid in question. A higher transport affinity for one amino acid may therefore lead to competitive inhibition of uptake of another amino acid. This concept was experimentally confirmed, with alanine and glycine being able to compete with ACC for uptake by LHT1, to reduce the ACC-induced triple response phenotype (Shin et al., 2015). Besides differences in uptake, we also observed differences in tissue specific accumulation between ACC and di-ACC, with the di-ACC much more restricted to the vascular tissues (Figure 2C). This is also likely due to a lower affinity of di-ACC for transporters, restricting movement of di-ACC *in planta*.

## ACC dimer evokes ethylene responses *via* ACO promiscuity

Our results show that the triple response phenotype observed in di-ACC treated plants (Figures 1B–D) is dependent on ethylene signaling (Figures 2A,B), and not ethylene-independent ACC signaling (Figure 3). We also show that di-ACC can be used for ethylene production both *in planta* (Figure 1G) and *in vitro* (Figure 4A), suggesting that ACOs are able to use di-ACC directly as substrate, rather than requiring prior peptidase activity to release ACC.

In the early days of ACO research, the specific ACO activity was sometimes questioned because non-enzymatic, oxidative breakdown of ACC to ethylene is possible under certain conditions. To control for this, early studies made use of 1-amino-2-ethylcyclopropane1-carboxylic acid (AEC) enantiomers, an ACC-related molecule which can be converted to 1-butene by ACO activity (McKeon and Shang Fa, 1984). While non-enzymatic conversion of AEC to 1-butene can make use of any AEC stereoisomer, ACOs primarily convert the 1R, 2S enantiomer (McKeon and Shang Fa, 1984; Wang and Yang, 1987; Nilsen et al., 1988; Ververidis and John, 1991; Dunkley and Golden, 1998). As an alternate substrate for ACO, AEC competes with ACC, reducing ethylene production (McKeon and Shang Fa, 1984). Furthermore, several other closely related ACC analogous molecules have been discovered to function either as competitive inhibitors of ACO activity (such as cyclopropane-1,1-dicarboxylic acid or 2-methylcyclopropanecarboxylic acid), leading to a reduced ethylene biosynthesis (Dourtoglou et al., 1999; Depaepe et al., 2020), while other molecules function as substrate mimics (such as cyclopropanecarboxylic acid), leading to an enhanced ethylene production (Dunkley and Golden, 1998; Dourtoglou et al., 1999). Together, these studies suggest that ACOs are promiscuous, and are able to use different substrates for ethylene synthesis with a lower efficiency than ACC. We now show that the ACC dipeptide represents a novel substrate for ACO-catalyzed ethylene synthesis (Figure 4A), although with a lower efficiency compared to ACC. Di-ACC is able to dock the catalytic site of ACO (Figure 4B), yet with a weaker affinity. This might explain why higher concentrations of di-ACC were required to give a similar phenotypic response as ACC, besides the effect of reduced uptake and mobility *in planta*.

## Conclusions

Dipeptides are important metabolic and/or signaling molecules in plants. The dipeptide of the non-proteinogenic amino acid ACC, which is also the precursor of ethylene biosynthesis, is taken up by Arabidopsis seedlings *via* LHT1 in a similar way as ACC. Di-ACC is predominantly confined to vascular tissue and turned into ethylene gas *via* substrate promiscuity of ACC-oxidase, albeit with a lower efficiency. This way, the ACC dimer is able to trigger ethylene production and thus forms an alternative compound for ACC to evoke weak ethylene responses. We also showed that di-ACC is not evoking ACC-specific ethylene independent responses, and that di-ACC is not naturally detected in Arabidopsis hypocotyl sections. If this di-ACC or similar ACC-conjugated peptides exist in other species remains to be discovered.

## Data availability statement

The original contributions presented in the study are included in the article/Supplementary material, further inquiries can be directed to the corresponding author.

## Author contributions

JV-H, AR, SR, and JP performed experimental work. DL performed the Marchantia experiments. ST, HS, YM, and YO performed the nano-PALDI MSI experiments. AV and BV supervised the project. JV-H and BV wrote the manuscript. All authors contributed to the article and approved the submitted version.

## Funding

This work was supported by a Research Foundation Flanders (FWO) Grant (G0G0219N) to BV and a PhD fellowship (1150822N) to JP, and a KU Leuven Grant (C14/18/056) to BV.

## Conflict of interest

The authors declare that the research was conducted in the absence of any commercial or financial relationships that could be construed as a potential conflict of interest.

## Publisher's note

All claims expressed in this article are solely those of the authors and do not necessarily represent those of their affiliated organizations, or those of the publisher, the editors and the reviewers. Any product that may be evaluated in this article, or claim that may be made by its manufacturer, is not guaranteed or endorsed by the publisher.
