# Supplementary material for: A 1-aminocyclopropane-1-carboxylic-acid (ACC) dipeptide elicits ethylene responses through ACC-oxidase mediated substrate promiscuity
Source: Front Plant Sci. 2022 Sep 12;13:995073. doi: 10.3389/fpls.2022.995073 (PMC9510837; doi:10.3389/fpls.2022.995073)
Supplement: Supplementary file 1 [file Data_Sheet_1.docx]

Supplemental Material

**A 1-aminocyclopropane-1-carboxylic-acid (ACC) dipeptide elicits ethylene responses through ACC-oxidase mediated substrate promiscuity**

John Vaughan-Hirsch^1^, Dongdong Li^1,2^, Albert Roig Martinez^3^, Stijn Roden^1^, Jolien Pattyn^1^, Shu Taira^4^, Hitomi Shikano^4^, Yoko Miyama^4^, Yukari Okano^4^, Arnout Voet^3^, Bram Van de Poel^1,5*^

^1^ Division of Crop Biotechnics, Department of Biosystems, University of Leuven, Willem de Croylaan 42, 3001 Leuven, Belgium

^2^ College of Agriculture and Biotechnology, Zhejiang University, Zijingang Campus, Hangzhou 310058, People’s Republic of China

^3^ Division of Biochemistry, Molecular and Structural Biology, Department of Chemistry, University of Leuven, Celestijnlaan 200G, 3001 Leuven, Belgium

^4^ Fukushima University, Department of Agriculture, 1-Kanayagawa, Fukushima 960–1296, Japan

^5^ KU Leuven Plant Institute, University of Leuven, Arenbergpark 30, 3001 Leuven, Belgium

Supplemental Figure S1

**
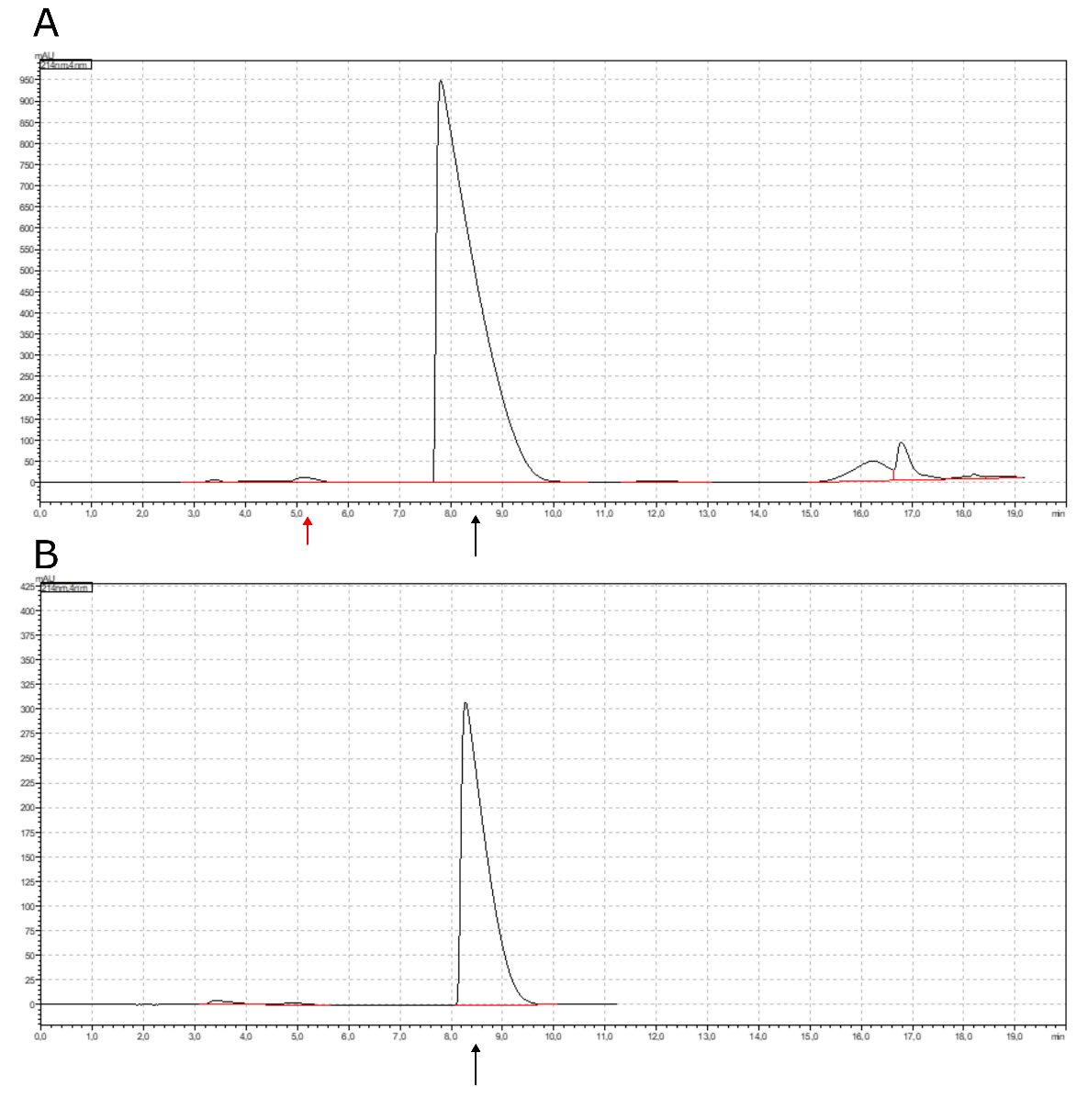
**

Figure S1: Di-ACC was purified by HPLC. Di-ACC stock before (A) and after (B) purification by HPLC. Black arrows indicate the di-ACC peak, the red arrow indicates the small contaminating peak at the retention time of ACC. Although the retention time for Fmoc-ACC is not known, only the clean di-ACC peak was collected (between retention time 7.5 – 10 min), eliminating any other contaminants.

Supplemental Figure S2

**
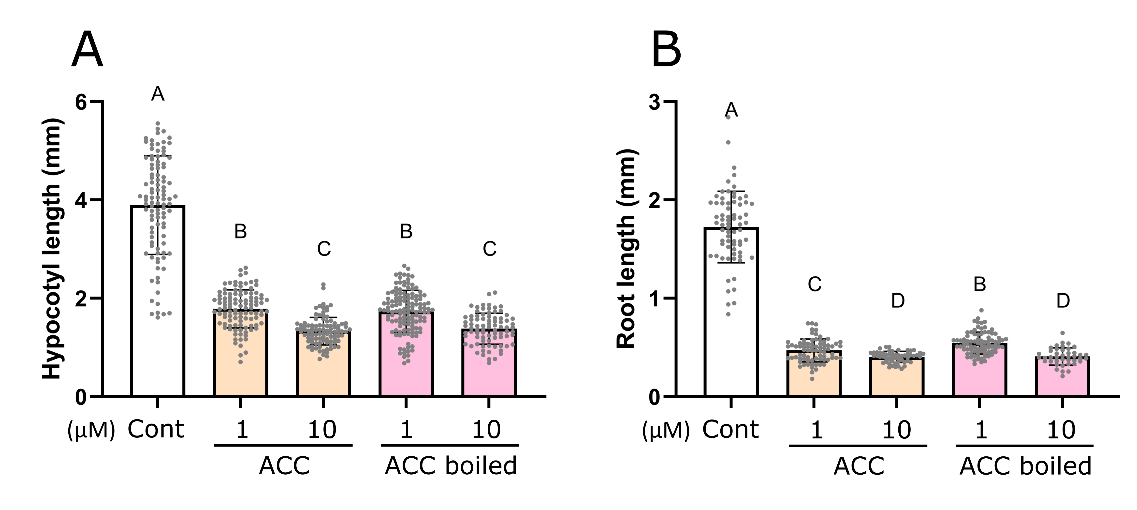
**

Figure S2: ACC stability is not affected by boiling. Hypocotyl (A) and root (B) length of 3 day-old dark-grown Arabidopsis seedlings after growth on ACC, or boiled ACC (for 1h) at the concentrations indicated.
